# Predictive Value of Inflammatory and Nutritional Indexes in the Pathology of Bladder Cancer Patients Treated with Radical Cystectomy

**DOI:** 10.3390/curroncol30030197

**Published:** 2023-02-21

**Authors:** Nebojsa Prijovic, Miodrag Acimovic, Veljko Santric, Branko Stankovic, Predrag Nikic, Ivan Vukovic, Ivan Soldatovic, Djordje Nale, Luka Kovacevic, Petar Nale, Adrian Marinkovic, Uros Babic

**Affiliations:** 1 Clinic of Urology, University Clinical Center of Serbia, Resavska Str. 51, 11000 Belgrade, Serbia; 2 Faculty of Medicine, University of Belgrade, Dr Subotica Str. 8, 11000 Belgrade, Serbia

**Keywords:** bladder cancer, pathology, inflammation, nutrition, geriatric nutritional risk index, systemic inflammatory response index, derived neutrophil-to-lymphocyte ratio

## Abstract

In recent years, the focus of numerous studies has been the predictive value of inflammatory and nutritional parameters in oncology patients. The aim of our study was to examine the relationship between the inflammatory and nutritional parameters and the histopathological characteristics of patients with bladder cancer. A retrospective study included 491 patients who underwent radical cystectomy for bladder cancer between 2017 and 2021. We calculated the preoperative values of the neutrophil-to-lymphocyte ratio (NLR), the derived neutrophil-to-lymphocyte ratio (dNLR), the systemic immune-inflammation index (SII), the systemic inflammatory response index (SIRI), the platelet-to-lymphocyte ratio (PLR), the lymphocyte-to-monocyte ratio (LMR), the prognostic nutritional index (PNI), and the geriatric nutritional risk index (GNRI). Statistically significant positive correlations were observed between NLR, dNLR, SII, SIRI, and PLR and the pathological stage of the tumor. We observed statistically significant inverse correlations for LMR, PNI, and GNRI with the tumor stage. SIRI was identified as an independent predictor of the presence of LVI. dNLR was identified as an independent predictor of positive surgical margins. GNRI was identified as an independent predictor of the presence of metastases in the lymph nodes. We noticed the predictive value of SIRI, dNLR, and GNRI in the pathology of bladder cancer patients.

## 1. Introduction

Bladder cancer (BC), with approximately 500,000 newly diagnosed cases worldwide, is the 10th most common cancer in the population. In the male population, bladder cancer occurs more often and ranks 6th in frequency among malignancies, with an incidence of 9.5 per 100,000 inhabitants [1]. In relation to geographic localization, there are variations in morbidity and mortality due to differences in exposure to risk factors as well as differences in diagnosis and the types of treatment that are available, so a higher incidence of bladder cancer has been observed in Western countries [2].

Bladder cancer primarily occurs in the elderly population, and the diagnosis is most often made between the ages of 70 and 84 [3]. It occurs more often in men (up to 3–4 times more), which can be explained by differences in lifestyle as well as by the possible retention of urine containing carcinogens in the field of prostate enlargement and urine retention [4]. Although it is diagnosed more often in men, it has been observed that BC manifests as a more aggressive disease in women [5]. The most significant risk factor for the occurrence of BC is tobacco smoking [6]. The second most common risk factor for the occurrence of BC is professional exposure to chemical agents containing polycyclic aromatic hydrocarbons and aromatic amines, which occurs most often when working with paints [7].

In about 90% of cases, BC is histopathological urothelial carcinoma, where pure urothelial carcinoma is present in about 75% of cases and a “variant” histology is present in 25% of cases [8]. Depending on the depth of the invasion, BC can be non-muscle-invasive BC (NMIBC) or muscle-invasive (MIBC), which is of crucial importance for the treatment method. BC is presented as NMIBC in about 70% of cases, and depending on the level of risk, the possibility of progression to MIBC is up to 60% [9]. In patients who experience disease progression to MIBC, the prognosis is worse than in those with primary MIBC [10]. While the basis of treatment for NMIBC is the transurethral resection of the BC tumor and the prevention of recurrence, the standard of care for localized MIBC and high-risk patients with NMIBC is radical cystectomy [11,12].

The identification of prognostic markers is of great importance in oncology. In BC after radical cystectomy, the most significant histopathological prognostic factors are the tumor stage and lymph node status, and the presence of lymphovascular invasion (LVI) was also recognized as a significant prognostic factor [13,14]. The treatment outcomes and the occurrence of postoperative complications in oncology patients are often related to their nutritional status, which can be impaired by cancer-induced chronic inflammation [15]. In a large number of cancers, it has been shown that biomarkers of the inflammatory response can predict the prognosis and outcomes of these patients [16,17,18,19]. In recent years, the focus of numerous studies with oncology patients has been markers that can be calculated from blood count parameters such as the neutrophil-to-lymphocyte ratio (NLR) [20,21], the derived neutrophil-to-lymphocyte ratio (dNLR), the systemic immuno-inflammatory index (SII), and the systemic inflammatory response index (SIRI) [19,22,23].

Tumor cells can produce colony-stimulating factors, which can subsequently lead to an increase in the number of leukocytes [24]. The roles of blood cells in the proliferation of tumor cells as well as their migration and invasion are also known [25,26]. This influence is explained by the production of cytokines, the suppression of peripheral T lymphocytes, the stimulation of angiogenesis, and the repair of DNA damage [27]. Moreover, studies have shown that there is an association between the immune response and tumor prognosis [28]. Since cancers are chronic debilitating diseases, the course of the disease itself and systemic inflammation can lead to malnutrition. Malnutrition in cancer patients is associated with immune suppression, which contributes to tumor progression and the creation of a favorable environment for tumor recurrence [29,30,31]. 

Among the indexes that indicate the nutritional and inflammatory statuses of a patient, the prognostic nutritional index (PNI) stands out. Its concept as a potential biomarker in gastrointestinal surgery was introduced by Buzby et al. [32]. The calculation of PNI is based on the level of serum albumin and the number of peripheral lymphocytes in the blood, and it is known that hypoalbuminemia and a reduced number of lymphocytes are associated with worse outcomes in patients with cancer [33,34]. One of the parameters that can be used to describe the nutritional status of oncology patients, especially those in whom the disease occurs at a later age, is the geriatric nutritional risk index (GNRI) [35]. GNRI is calculated based on serum albumin levels, body mass, and body height and is considered more reliable for assessing nutritional status than body mass index (BMI) and serum albumin alone in the elderly patient population [36].

Given the relative lack of data on the association of inflammatory and nutritional parameters with the characteristics of invasive BC, the aim of our study was to examine the association of these parameters with the histopathological characteristics of BC in patients treated with radical cystectomy.

## 2. Materials and Methods

### 2.1. Screening Cohort and Baseline Characteristics

This retrospective single-center study included patients who underwent radical cystectomy for primary bladder cancer during the period from 1 January 2017 to 31 December 2021 at the Urology Clinic of the University Clinical Center of Serbia in Belgrade. The study included patients undergoing radical cystectomy for previously histopathologically verified MIBC or very high risk NMIBC in clinical stage cT2-T4 without the presence of clinically verified preoperative lymph node metastasis (cN0) or distant metastasis (cM0). The study did not include patients undergoing salvage cystectomy who had previously been clinically verified to have metastases or patients with secondary bladder malignancies. Moreover, the study did not include patients with hematological diseases and immunodeficiency conditions characterized by disturbances in the blood count.

Data on demographic and clinical characteristics (gender, age, body weight, body height, body mass index, smoking, and Eastern Cooperative Oncology Group (ECOG) performance status), laboratory results (numbers of leukocytes, neutrophils, lymphocytes, and monocytes; hemoglobin value; platelet count; and albumin level in peripheral blood), and histopathological characteristics (type of cancer, tumor stage, presence of lymphovascular invasion, status of surgical margins, and status of lymph nodes) were taken from the patients’ medical records. Blood samples were taken immediately after admission and were analyzed in the laboratory of the Center for Medical Biochemistry of the University Clinical Center of Serbia in Belgrade. The histopathological diagnoses were determined by an experienced uropathologist. The BC type was determined according to the current World Health Organization classification [37]. The tumor stage was determined according to the current TNM classification of bladder cancer [38]. 

### 2.2. Inflammatory and Nutritional Index Calculations

We calculated NLR as neutrophil count (×10^9^/L)/lymphocyte count (×10^9^/L). dNLR was calculated as neutrophil count (×10^9^/L) / (leukocyte count (×10^9^/L)—neutrophil count (×10^9^/L)). SII was calculated as platelet count (×10^9^/L) × neutrophil count (×10^9^/L)/lymphocyte count (×10^9^/L). SIRI was calculated as neutrophil count (×10^9^/L) × monocyte count (×10^9^/L)/ lymphocyte count (×10^9^/L). The ratio of lymphocytes to monocytes (LMR) was calculated as lymphocyte count (×10^9^/L) / monocyte count. The platelet-to-lymphocyte ratio (PLR) was calculated as platelet count (×10^9^/L) / lymphocyte count (×10^9^/L). PNI was calculated according to the formula PNI = serum albumin (g/L) + 5 × total lymphocyte count (×10^9^/L). We calculated GNRI as 1.487 × serum albumin (g/L) + 41.7 × body mass (kg)/ideal body mass (kg). We calculated the ideal body mass as 22× body height^2^ (m^2^). 

### 2.3. Statistical Analysis

Descriptive and analytical statistics methods were used for statistical data processing. The Kolmogorov–Smirnov test was used to test the normal distribution of the data. The significance of the difference of variables with normal distributions was analyzed using Student’s *t*-test for two independent samples, while for variables that did not have normal distributions, the Mann–Whitney U test was used. The existence of correlations between variables was determined using Spearman’s rank correlation. Variables for which a statistically significant difference was observed were analyzed using a binary logistic regression analysis (in a model that included gender, age, and ECOG status) in order to identify independent predictors of certain histopathological properties. A value of *p* < 0.05 was considered statistically significant. SPSS version 20 for Windows was used for statistical data processing.

## 3. Results

In the period from 1 January 2017 to 31 December 2021, a total of 491 patients who met the criteria for inclusion in this study underwent radical cystectomy for primary localized or locally advanced BC. The demographic, clinical, and histopathological characteristics of the patients are presented in Table 1. Of the total number, 387 (78.8%) patients were men, while 104 (21.2%) were women. The median age was 67 years, with an interquartile range (IQR) of 62–72 years. Observing the ECOG performance status, the largest number (242; 49.3%) had a score of 1, 202 (41.4%) of them had a score of 0, 40 (8.1%) had a score of 2, and 7 (1.4%) had a score of 3. In the histopathological findings after radical cystectomy, the pT0 stage was recorded in 13 (2.6%) patients, pTa and pTis were recorded in 12 (12.4%), pT1 was recorded in 37 (7.5%), pT2 was recorded in 160 (32.6%), pT3 was recorded in 154 (31.4 %), and pT4 was recorded in 115 (23.4%) patients. Observing the presence of tumor invasion of the muscle layer, NMIBC was present in 49 (10.3%) patients, while MIBC was present in 429 (89.7%). In 95.4% of cases, urothelial carcinoma was present. Lymphovascular invasion was observed in 334 (70.6%) cases. The surgical margins were positive in 76 (15.5%) patients. The lymph nodes were positive in 81 (23.2%) patients who underwent pelvic lymphadenectomy.

Table 2 presents the values of the laboratory, inflammatory, and nutritional indexes. The median NLR was 2.68 (IQR 84–3.80), and the median dNLR was 1.71 (IQR 1.26–2.38). The median SII was 638.08 (IQR 420.00–1032.77), and the median SIRI was 1.53 (IQR 0.97–2.45). The median LMR was 3.08 (IQR 2.26–4.00), while the median PLR was 135.90 (IQR 107.27–190.00). The observed median PNI was 49.00 (IQR 45.00–53.00), and the median GNRI was 107.99 (IQR 100.95–115.33).

The results of examining the correlations between the inflammatory and nutritional indexes and the tumor stage are shown in Table 3. Statistically significant positive correlations were observed between NLR (*p* < 0.001), dNLR (*p* < 0.001), SII (*p* < 0.001), SIRI (*p* < 0.001), and PLR. (*p* < 0.001) and the pathological stage of the tumor. We observed statistically significant inverse correlations of LMR (*p* = 0.001), PNI (*p* < 0.001), and GNRI (*p* = 0.001) with the pathological stage of the tumor.

The associations of inflammatory and nutritional parameters with muscle invasion are displayed in Table 4. The values of NLR (*p* = 0.004), dNLR (*p* = 0.01), SII (*p* = 0.007) and SIRI (*p* = 0.006)) were significantly higher in patients with MIBC, while the values of LMR (*p* = 0.04) and GNRI (*p* = 0.033) were significantly lower in patients with MIBC. 

Comparing the values of the examined parameters among patients with urothelial cancer and other types of bladder cancer (Table 5), the SIRI value was significantly higher in patients with other histological types of cancer (*p* = 0.042).

Table 6 presents the results of the comparison of the inflammatory and nutritional parameters with the presence of lymphovascular invasion. The values of NLR (*p* = 0.002), dNLR (*p* = 0.009), SII (*p* = 0.002), and SIRI (*p* = 0.001) were significantly higher in cases where LVI was present. The values of PNI (*p* = 0.003) and GNRI (*p* = 0.004) were significantly lower in patients in whom LVI was present.

The associations of inflammatory and nutritional parameters with the status of the surgical margins are shown in Table 7. The values of NLR (*p* = 0.02), dNLR (*p* = 0.007), and SII (*p* = 0.037) were significantly higher in patients with positive surgical margins.

Table 8 shows the relationships between the examined parameters and the status of the lymph nodes. The values of NLR (*p* = 0.036), dNLR (*p* = 0.01), and SII (*p* = 0.022) were significantly higher in patients with positive lymph nodes, while the values of GNRI (*p* = 0.026) were significantly lower in these patients.

Table 9 shows the results of the binary logistic regression analysis of predictors of histopathological characteristics of BC. None of the examined parameters were identified as independent predictors of muscle layer invasion. Age was observed as a predictive factor of the histological type of BC (*p* = 0.034). SIRI was identified as an independent predictor (*p* = 0.045) of the presence of LVI. Female gender (*p* = 0.001) and dNLR (*p* = 0.032) were identified as independent predictors of positive surgical margins among the examined parameters. GNRI was identified as an independent predictor of the presence of metastases in the lymph nodes (*p* = 0.026).

**Figure 1:**
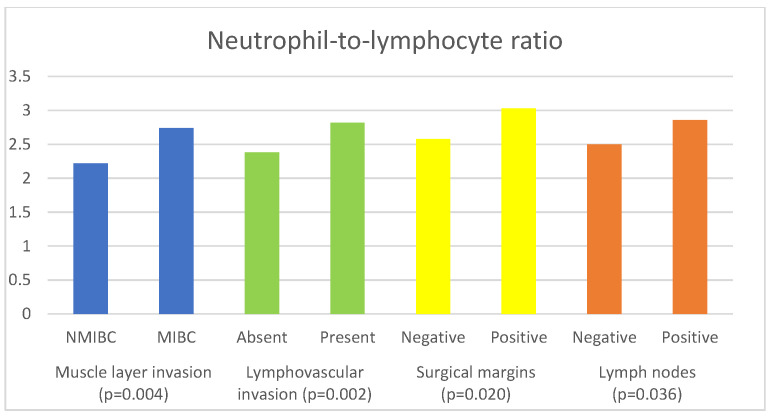
Significant associations of neutrophil-to-lymphocyte ratio with histopathological characteristics of bladder cancer.

Figure 1 shows the significant associations of NLR with the histopathological characteristics of BC.

**Figure 2:**
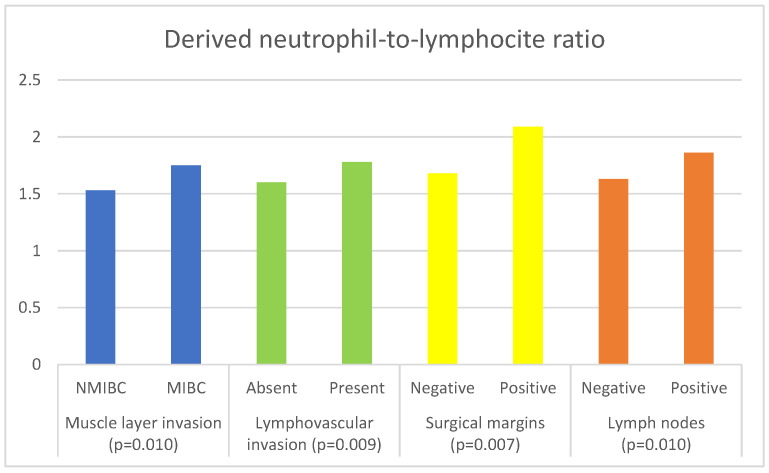
Significant associations of derived neutrophil-to-lymphocyte ratio with histopathological characteristics of bladder cancer.

The significant associations of dNLR with the histopathological characteristics are shown graphically in Figure 2.

**Figure 3:**
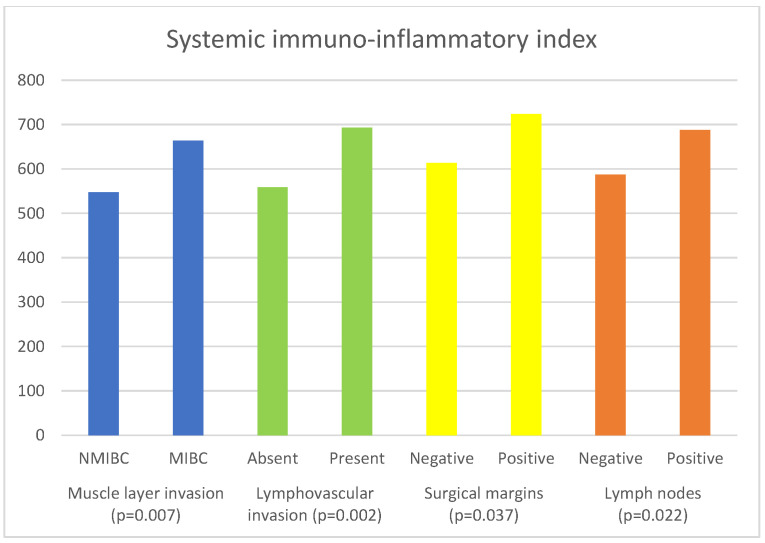
Significant associations of systemic immuno-inflammatory index with histopathological characteristics of bladder cancer.

Figure 3 shows the significant associations of SII with the histopathological characteristics of BC.

**Figure 4:**
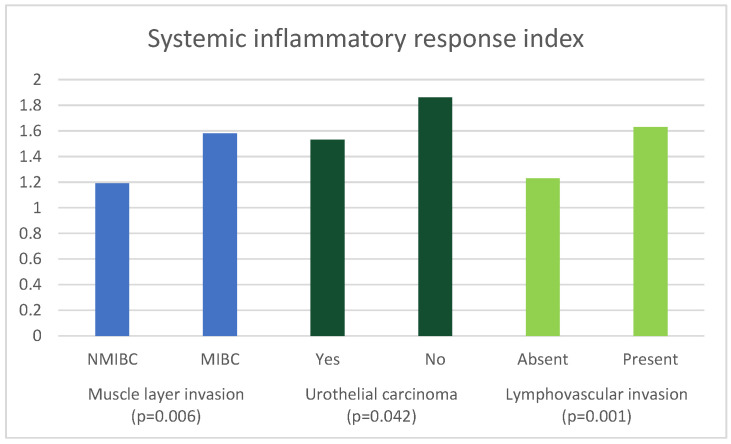
Significant associations of systemic inflammatory response index with histopathological characteristics of bladder cancer.

A graphical presentation of the significant associations of SIRI with the histopathological features of BC is presented in Figure 4.

**Figure 5:**
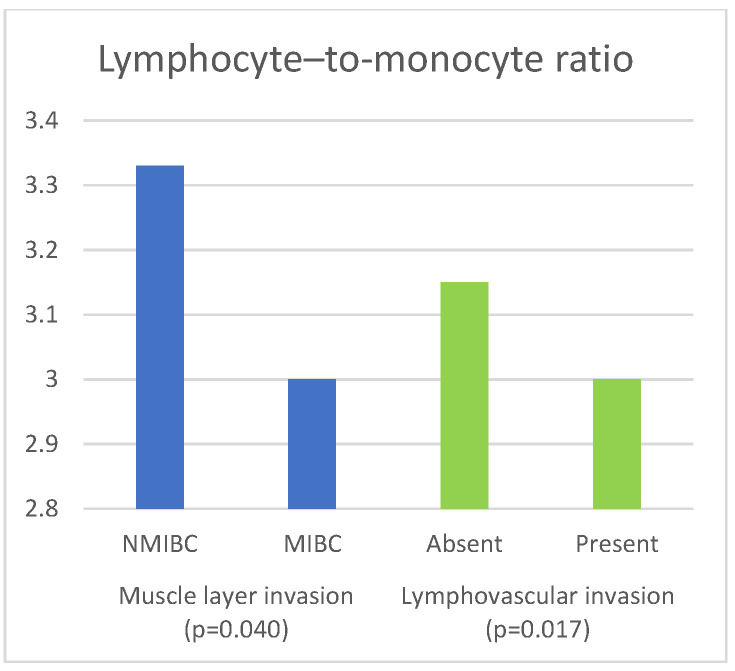
Significant associations of lymphocyte-to-monocyte ratio with histopathological characteristics of bladder cancer.

The significant associations of LMR with the histopathological characteristics of bladder cancer are shown in Figure 5.

**Figure 6:**
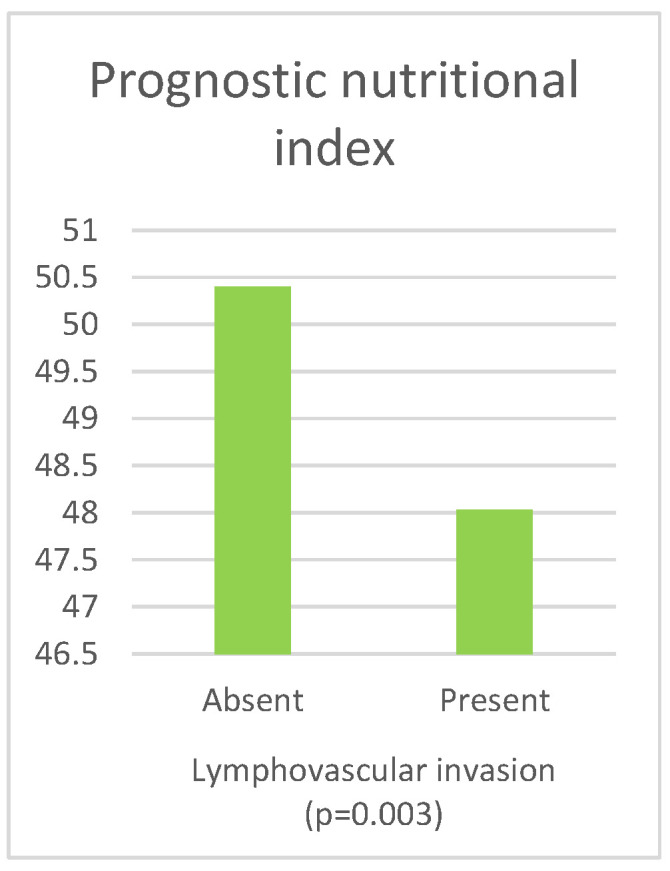
Significant difference in PNI between patients with and without LVI.

A graphically displayed significant difference in PNI between patients with and without LVI is given in Figure 6.

**Figure 7:**
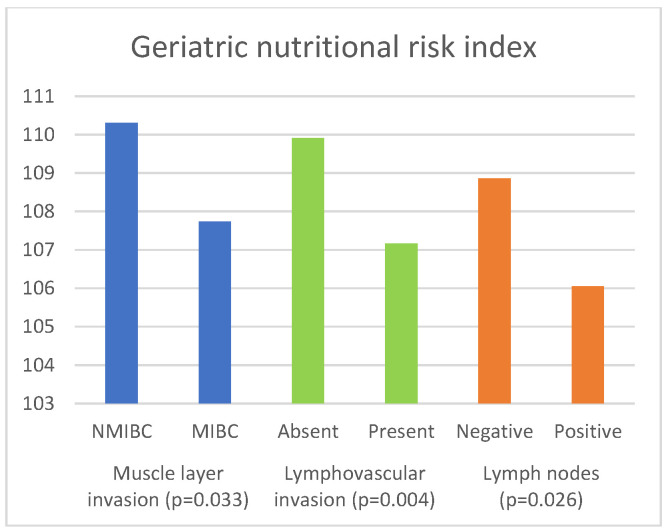
Significant associations of geriatric nutritional risk index ratio with histopathological characteristics of bladder cancer.

Figure 7 presents the significant associations of GNRI with the histopathological characteristics of BC.

## 4. Discussion

Considering that the 5-year survival of patients with MIBC is below 50% [39], there is a need to discover biomarkers that could be linked with the prognosis of the disease. It is widely accepted that a potential biomarker should be easily accessible. Hence, in the last few years, markers that can be calculated based on the complete blood count and routine biochemical analysis parameters, such as albumin, have been the focus of numerous studies in oncology. It was shown that inflammation plays a significant role in the biological behavior of tumors, and therefore it was considered a “seventh hallmark” of cancer [40]. Moreover, there is accumulating evidence connecting inflammation and nutrition with prognosis in various cancer types [41,42]. Considering that white blood cells and albumin reflect the inflammatory response and nutritional status, biomarkers that include these parameters can potentially indicate the characteristics of the tumor and the prognosis of the disease.

After radical cystectomy, the estimated pathological tumor (pT) stage is one of the most significant prognostic factors in patients with BC [13]. In our study, we found correlations between all examined inflammatory and nutritional parameters with the pT stage after radical cystectomy. Among the inflammatory parameters, positive correlations with the tumor stage were recorded for all, with the exception of LMR, which showed an inverse correlation. Furthermore, inverse correlations with the tumor stage after radical cystectomy were found for PNI and GNRI. Our results are comparable with the findings of Tang X et al., who showed higher values of the inflammatory parameters NLR, dNLR, PLR, and SII in patients with MIBC compared to patients with NMIBC, while LMR and PNI were lower in patients with MIBC [43]. Moreover, a recent Bi H study from 2020 also showed an association of lower PNI values with a higher pT stage in patients with high-risk NMIBC [44]. Concerning the nutritional parameters, lower values of PNI and GNRI, which indicate malnutrition, correlated with a higher pT stage in our study. To the best of our knowledge, there are no studies published so far examining the association of GNRI with the histopathological characteristics of BC. Moreover, when comparing patients with NMIBC and MIBC, we observed that NLR, dNLR, SII, and SIRI were significantly higher in patients with MIBC, while LMR and GNRI were significantly lower in this group. Nevertheless, we did not find any of the examined inflammatory and nutritional parameters to be independent predictors of muscle layer invasion, which may be explained by the predominantly MIBC patients included in this study (89.7%).

Different histologic types of BC and different histologic subtypes of urothelial cancer have different biological behaviors and prognoses [37]. Urothelial carcinoma (UC) is the most common histological type of BC in the developed world, with the pure urothelial cancer histology present in about 75% of cases and a “variant” histology present in 25% of cases [8]. Our results showed that SIRI was significantly higher in patients who had a diverse histology, i.e., non-urothelial BC.

The presence of lymphovascular invasion (LVI) is considered as prognostic factor of tumor aggressiveness and the possible existence of occult metastases in various types of cancers [45,46,47]. The presence of LVI in the histology findings after radical cystectomy in patients with MIBC is associated with more aggressive disease and is a predictive factor for survival [14]. In our study, NLR, dNLR, SII, and SIRI were all significantly higher in cases where LVI was present, while LMR, PNI, and GNRI were significantly lower in the same group of patients. Similar findings were observed by Bi et al., emphasizing the association of higher SII and lower PNI with the presence of LVI in a study conducted only in patients with NMIBC [44].

Accordingly, we identified SIRI as an independent predictor of the presence of LVI. The SIRI value is directly proportional to the number of neutrophils and monocytes and inversely proportional to the number of lymphocytes. The interpretation of our results is consistent with the evidence that neutrophils influence tumor progression by releasing elastase, which degrades the extracellular matrix and promotes neovascularization [48]. Moreover, a decrease in the number of lymphocytes leads to a decrease in local immunity, which creates an immunocompromised environment that favors tumor growth [49]. Given that elevated neutrophil counts and decreased lymphocyte counts create a favorable environment for local tumor progression, this could potentially explain the association of higher SIRI values with the presence of LVI observed in our study.

The importance of surgical margin status after radical cystectomy is still controversial. Studies that investigated the impact of positive surgical margins after radical cystectomy on survival showed conflicting results [50,51]. However, a meta-analysis by Hong X et al. stressed the association of positive surgical margins with worse overall survival after radical cystectomy [52]. Therefore, the potential preoperative prediction of the status of surgical margins may have significance in surgical practice. In this study, we found higher NLR, dNLR, and SII in patients with positive margins and identified dNLR as an independent predictor of positive surgical margins after radical cystectomy. Findings published in the literature indicate an association of elevated dNLR with more aggressive BC in terms of a higher grade and tumor stage [43]. Nevertheless, in patients with ovarian cancer, Wu et al. showed that dNLR values differ between patients with malignant and benign ovarian pathologies [53].

It has been reported previously that dNLR increases as a consequence of an increased number of neutrophils and a decreased number of other types of white blood cells, including lymphocytes, and such changes in the white blood cell count contribute to local tumor progression [48,49].

The presence of lymph node metastases is one of the most important histopathological prognostic factors after radical cystectomy [13]. Preoperatively, the assessment of lymph nodes using conventional imaging methods is still limited by the fact that metastases cannot be detected in normal-sized or minimally enlarged lymph nodes [12,54,55,56]. Given that the presence of lymph node metastases after radical cystectomy is associated with poor prognosis and worse overall survival [13], the identification of biomarkers that could predict the presence of micrometastases in clinically normal lymph nodes would be of great importance in the selection of patients with BC for radical cystectomy. In our research, we found significantly higher NLR, dNLR, and SII in patients with positive LN, while in the same group the values of GNRI were significantly lower. Moreover, we identified GNRI as an independent predictor of the presence of metastasis in surgically resected lymph nodes after radical cystectomy. According to the literature data, GNRI has been established as an index to predict morbidity and mortality among elderly patients, with lower values indicating malnutrition and being associated with a higher risk of death [35,36]. Hence, we chose GNRI for the purpose of this study because of the age of our patients (median age of 67 years, IQR 62–72). Nevertheless, this index is considered most suitable in the elderly patient population because it is calculated based on albumin values and an ideal body mass, unlike others that include a normal body weight [57,58]. In recent years, this index has been used as a parameter for the prognosis of various chronic diseases and malignancies [59,60].

To the best of our knowledge, no studies examining the association between GNRI and the histopathological features of BC were published so far. Nonetheless, in previously published studies in patients with other genito-urinary cancers, GNRI was recognized as a prognostic predictor in patients with non-metastatic renal cell carcinoma and in metastatic hormone-naive prostate carcinoma [61,62]. Considering that we have identified GNRI as an independent predictor of the presence of metastases in the lymph nodes, there is room for further research into the significance of this index in patients with BC.

Our study had several limitations. First, it was a retrospective observational study. In addition, the study was conducted at a single institution. Moreover, a limitation of the study was the relatively small number of patients.

## 5. Conclusions

Our study showed multiple associations of inflammatory and nutritional indexes with the histopathological characteristics of urothelial bladder carcinoma. Moreover, we found predictive value of SIRI, dNLR, and GNRI for the presence of lymphovascular invasion, positive surgical margins, and the presence of lymph node metastases after radical cystectomy, respectively.

Considering that these easily accessible biomarkers could potentially predict the histopathological features of poor prognosis, they might be used to improve patient selection for radical surgical treatment. In order to further explore the predictive value of inflammatory and nutritional indexes in patients with bladder cancer, it would be necessary to conduct a prospective study with a larger number of patients and investigate the influences of these parameters on patient survival.

## Figures and Tables

**Table 1:** Demographic, clinical, and histopathological characteristics of patients.

Characteristics	Total
Patients, *n*	491
Gender, *n* (%)	
Male	387 (78.8%)
Female	104 (21.2%)
Age, median (IQR)	67.00 (62.00–72.00)
Body mass index, median (IQR)	25.74(23.44–28.08)
Tobacco smoking, *n* (%)	
No	158 (32.2%)
Current/former smoker	333 (67.8%)
ECOG, *n* (%)	
0	202 (41.1%)
1	242 (49.3%)
2	40 (8.1%)
3	7 (1.4%)
Pathological stage, *n* (%)	
pT0	13 (2.6%)
pTa + pTis	12 (2.4%)
pT1	37 (7.5%)
pT2	160 (32.6%)
pT3	154 (31.4%)
pT4	115 (23.4%)
Muscle invasion, *n* (%)	
NMIBC	49 (10.3%)
MIBC	429 (89.7%)
Histological type, *n* (%)	
Urothelial cancer (UC)	456 (95.4%)
Other type of cancer	22 (4.6%)
Lymphovascular invasion, *n* (%)	
Present	334 (70.6%)
Absent	139 (29.4%)
Surgical margins, *n* (%)	
Positive	76 (15.5%)
Negative	415 (84.5%)
Lymph nodes, *n* (%)	
Positive	81 (23.2%)
Negative	268 (76.8%)

**Table 2:** Values of laboratory, nutritional, and inflammatory parameters.

Parameter	Median (IQR)
Hemoglobin (g/L)	126.00 (107.00–141.00)
Leukocytes (×10^9^/L)	7.60 (6.20–9.20)
Neutrophils (×10^9^/L)	4.70 (3.60–6.10)
Lymphocytes (×10^9^/L)	1.80 (1.40–2.25)
Monocytes (×10^9^/L)	0.60 (0.47–0.75)
Platelets (×10^9^/L)	245.00 (203.00–307.00)
NLR	2.68 (1.84–3.80)
dNLR	1.71(1.26–2.38)
SII	638.08 (420.00–1032.77)
SIRI	1.53 (0.97–2.45)
LMR	3.08 (2.26–4.00)
PLR	135.90 (107.27–190.00)
Albumin (g/L)	40.00 (37.00–43.00)
PNI	49.00 (45.00–53.00)
GNRI	107.99 (100.95–115.33)

NLR, neutrophil-to-lymphocyte ratio; dNLR, derived neutrophil-to-lymphocyte ratio; SII, systemic immuno-inflammatory index; SIRI, systemic inflammatory response index; LMR, lymphocyte-to-monocyte ratio; PLR, platelet-to-lymphocyte ratio; PNI, prognostic nutritional index; GNRI, geriatric nutritional risk index.

**Table 3:** Correlations between the inflammatory and nutritional parameters and the pathological tumor stage.

Parameter	Correlation Coefficient ^a^	*p* Value
NLR	0.225	<0.001
dNLR	0.228	<0.001
SII	0.234	<0.001
SIRI	0.227	<0.001
LMR	−0.152	0.001
PLR	0.174	<0.001
PNI	−0.182	<0.001
GNRI	−0.155	0.001

^a^ Spearman’s rank correlation. NLR, neutrophil-to-lymphocyte ratio; dNLR, derived neutrophil-to-lymphocyte ratio; SII, systemic immuno-inflammatory index; SIRI, systemic inflammatory response index; LMR, lymphocyte-to-monocyte ratio; PLR, platelet-to-lymphocyte ratio; PNI, prognostic nutritional index; GNRI, geriatric nutritional risk index.

**Table 4:** Associations of inflammatory and nutritional parameters with muscle layer invasion.

Parameter	Muscle Invasion	Median (IQR)	*p* Value
NLR	NMIBC	2.22 (1.55–2.92)	0.004 ^a^
	MIBC	2.74 (1.87–3.92)	
dNLR	NMIBC	1.53 (1.22–1.89)	0.010 ^a^
	MIBC	1.75 (1.29–2.44)	
SII	NMIBC	547.62 (340.20–739.6	0.007 ^a^
	MIBC	663.88 (441.37–1074.73)	
SIRI	NMIBC	1.19 (0.76–1.97)	0.006 ^a^
	MIBC	1.58 (1.01–2.53)	
LMR	NMIBC	3.33 (2.40–4.25)	0.040 ^a^
	MIBC	3.00 (2.25–4.00)	
PLR	NMIBC	125.88 (97.92–165.12)	0.075 ^a^
	MIBC	137.83 (108.30–195.06)	
PNI	NMIBC	51.00 (47.50–54.60)	0.062 ^b^
	MIBC	48.50 (45.00–52.80)	
GNRI	NMIBC	110.31 (103.37–115.99)	0.033 ^a^
	MIBC	107.74 (100.87–114.97)	

^a^ Mann–Whitney U test, ^b^ Student’s *t* test; NLR, neutrophil-to-lymphocyte ratio; dNLR, derived neutrophil-to-lymphocyte ratio; SII, systemic immuno-inflammatory index; SIRI, systemic inflammatory response index; LMR, lymphocyte-to-monocyte ratio; PLR, platelet-to-lymphocyte ratio; PNI, prognostic nutritional index; GNRI, geriatric nutritional risk index.

**Table 5:** Associations of inflammatory and nutritional parameters with the histological type of bladder cancer.

Parameter	Urothelial Carcinoma	Median (IQR)	*p* Value
NLR	Yes	2.67 (1.83–3.75)	0.105 ^a^
	No	3.14 (2.16–4.08)	
dNLR	Yes	1.73 (1.26–2.37)	0.176 ^a^
	No	1.94 (1.59–2.45)	
SII	Yes	635.50 (423.41–1027.10)	0.283 ^a^
	No	774.86 (490.50–1093.47)	
SIRI	Yes	1.53 (0.98–2.40)	0.042 ^a^
	No	1.86 (1.24–3.24)	
LMR	Yes	3.08 (2.31–4.00)	0.104 ^a^
	No	2.37 (1.75–3.75)	
PLR	Yes	135.89 (107.48–189.09)	0.744 ^a^
	No	151.31 (108.89–195.26)	
PNI	Yes	49.00 (45.00–53.00)	0.428 ^a^
	No	48.78 (45.50–53.20)	
GNRI	Yes	107.84 (100.93–115.04)	0.697 ^b^
	No	108.55 (101.18–117.77)	

^a^ Mann–Whitney U test, ^b^ Student’s *t* test; NLR, neutrophil-to-lymphocyte ratio; dNLR, derived neutrophil-to-lymphocyte ratio; SII, systemic immuno-inflammatory index; SIRI, systemic inflammatory response index; LMR, lymphocyte-to-monocyte ratio; PLR, platelet-to-lymphocyte ratio; PNI, prognostic nutritional index; GNRI, geriatric nutritional risk index.

**Table 6:** Associations of inflammatory and nutritional parameters with the presence of lymphovascular invasion.

Parameter	Lymphovascular Invasion	Median (IQR)	*p* Value
NLR	Absent	2.38 (1.63–3.26)	0.002 ^a^
	Present	2.82 (1.93–4.09)	
dNLR	Absent	1.60 (1.20–2.09)	0.009 ^a^
	Present	1.78 (1.31–2.45)	
SII	Absent	558.54 (376.18–893.33)	0.002 ^a^
	Present	693.00 (450.78–1095.88)	
SIRI	Absent	1.23 (0.86–2.07)	0.001 ^a^
	Present	1.63 (1.02–2.57)	
LMR	Absent	3.15 (2.49–4.25)	0.017 ^a^
	Present	3.00 (2.20–4.00)	
PLR	Absent	132.50 (100.91–172.00)	0.161 ^a^
	Present	137.93 (108.57–197.50)	
PNI	Absent	50.40 (46.55–53.50)	0.003 ^b^
	Present	48.03 (44.50–52.50)	
GNRI	Absent	109.91 (104.29–116.16)	0.004 ^b^
	Present	107.17 (100.11–114.72)	

^a^ Mann–Whitney U test, ^b^ Student’s *t* test; NLR, neutrophil-to-lymphocyte ratio; dNLR, derived neutrophil-to-lymphocyte ratio; SII, systemic immuno-inflammatory index; SIRI, systemic inflammatory response index; LMR, lymphocyte-to-monocyte ratio; PLR, platelet-to-lymphocyte ratio; PNI, prognostic nutritional index; GNRI, geriatric nutritional risk index.

**Table 7:** Association of inflammatory and nutritional parameters with the status of surgical margins.

Parameter	Surgical Margin	Median	*p* Value
NLR	Negative	2.58 (1.79–3.69)	0.020 ^a^
	Positive	3.03 (2.21–4.29)	
dNLR	Negative	1.68 (1.24–2.31)	0.007 ^a^
	Positive	2.09 (1.48–2.58)	
SII	Negative	613.60 (409.09–1005.81)	0.037 ^a^
	Positive	723.67 (502.43–1221.01)	
SIRI	Negative	1.50 (0.97–2.40)	0.119 ^a^
	Positive	1.69 (1.19–3.13)	
LMR	Negative	3.11 (2.32–4.00)	0.659 ^a^
	Positive	3.00 (2.14–4.18)	
PLR	Negative	134.88 (106.94–186.11)	0.525 ^a^
	Positive	146.93 (107.87–197.08)	
PNI	Negative	49.00 (45.40–53.00)	0.440 ^b^
	Positive	48.28 (44.15–52.80)	
GNRI	Negative	108.40 (101.29–115.33)	0.328 ^b^
	Positive	105.57 (100.24–115.12)	

^a^ Mann–Whitney U test, ^b^ Student’s *t* test; NLR, neutrophil-to-lymphocyte ratio; dNLR, derived neutrophil-to-lymphocyte ratio; SII, systemic immuno-inflammatory index; SIRI, systemic inflammatory response index; LMR, lymphocyte-to-monocyte ratio; PLR, platelet-to-lymphocyte ratio; PNI, prognostic nutritional index; GNRI, geriatric nutritional risk index.

**Table 8:** Associations of inflammatory and nutritional parameters with the status of the lymph nodes.

Parameter	Lymph Nodes	Median (IQR)	*p* Value
NLR	Negative	2.50 (1.72–3.54)	0.036 ^a^
	Positive	2.86 (2.09–4.03)	
dNLR	Negative	1.63 (1.21–2.23)	0.010 ^a^
	Positive	1.86 (1.46–2.58)	
SII	Negative	587.32 (394.27–951.27)	0.022 ^a^
	Positive	687.50 (510.00–1108.42)	
SIRI	Negative	1.49 (0.94–2.36)	0.192 ^a^
	Positive	1.63 (1.02–2.56)	
LMR	Negative	3.13 (2.33–4.15)	0.613 ^a^
	Positive	3.12 (2.33–4.00)	
PLR	Negative	131.51 (101.12–174.07)	0.084 ^a^
	Positive	143.57 (117.00–195.26)	
PNI	Negative	49.45 (45.75–53.45)	0.351 ^b^
	Positive	48.50 (45.70–52.10)	
GNRI	Negative	108.86 (103.90–115.34)	0.026 ^b^
	Positive	106.05 (100.01–112.76)	

^a^ Mann–Whitney U test, ^b^ Student’s *t* test; NLR, neutrophil-to-lymphocyte ratio; dNLR, derived neutrophil-to-lymphocyte ratio; SII, systemic immuno-inflammatory index; SIRI, systemic inflammatory response index; LMR, lymphocyte-to-monocyte ratio; PLR, platelet-to-lymphocyte ratio; PNI, prognostic nutritional index; GNRI, geriatric nutritional risk index.

**Table 9:** Binary logistic regression analysis of independent predictors of bladder cancer histopathological characteristics.

Parameter	Muscle Layer Invasion		Histological Type		Lymphovascular Invasion		Positive Surgical Margins		Positive Lymph Nodes	
	OR (95% CI)	*p*	OR (95% CI)	*p*	OR (95% CI)	*p*	OR (95% CI)	*p*	OR (95% CI)	*p*
Gender (female)	0.693 (0.346–1.388)	0.301	1.449 (0.546–3.845)	0.456	1.060 (0.634–1.772)	0.825	2.544 (1.480–4.373)	0.001	1.443 (0.786–2.647)	0.237
Age	1.009 (0.964–1.056)	0.711	0.942 (0.891–0.996)	0.034	0.980 (0.950–1.012)	0.225	0.968 (0.934–1.002)	0.068	0.984 (0.947–1.022)	0.403
ECOG	1.472 (0.802–2.701)	0.212	1.344 (0.669–0.698)	0.406	1.332 (0.892–1.990)	0.160	1.247 (0.806–1.928)	0.321	1.065 (0.661–1.716)	0.796
NLR	0.946 (0.385–2.328)	0.904	-	-	1.201 (0.763–1.890)	0.428	0.796 (0.561–1.130)	0.202	0.915 (0.743–1.126)	0.400
dNLR	1.168 (0.313–4.349)	1.168	-	-	0.718 (0.373–1.380)	0.320	1.908 (1.058–3.443)	0.032	1.612 (0.997–2.607)	0.052
SII	1.001 (0.999–1.002)	0.426	-	-	1.000 (0.999–1.000)	0.534	1.000 (0.999–1.000)	0.760	1.000 (0.999–1.000)	0.444
SIRI	1.248 (0.742–2.098)	0.404	1.033 (0.937–1.139)	0.511	1.332 (1.006–1.765)	0.045	-	-	-	
LMR	1.064 (0.866–1.306)	0.554	-	-	1.135 (0.946–1.362)	0.127	-	-	-	
PNI	-	-	-	-	0.971 (0.919–1.027)	0.309	-	-	-	
GNRI	0.999 (0.968–1.030)	0.932	-	-	0.985 (0.959–1.012)	0.289	-	-	0.971 (0.945–0.996)	0.026

ECOG, Eastern Cooperative Oncology Group; NLR, neutrophil-to-lymphocyte ratio; dNLR, derived neutrophil-to-lymphocyte ratio; SII, systemic immuno-inflammatory index; SIRI, systemic inflammatory response index; LMR, lymphocyte-to-monocyte ratio; PNI, prognostic nutritional index; GNRI, geriatric nutritional risk index.

## Data Availability

The datasets used and analyzed during the current study are available from the corresponding author on reasonable request.
